# Supplementary material for: Antibody Profiling of Microbial Antigens in the Blood of COVID-19 mRNA Vaccine Recipients Using Microbial Protein Microarrays
Source: Vaccines (Basel). 2023 Nov 7;11(11):1694. doi: 10.3390/vaccines11111694 (PMC10674746; doi:10.3390/vaccines11111694)
Supplement: Supplementary file 1 [file vaccines-11-01694-s001.zip › Supplementary Table S5 Temporary changes of the microbial antibodies with highest sharp values.pdf]

Supplementary Table S5 Temporary changes of the microbial antibodies with highest SHAP values

	A				B				C				D				E				F				G				H				I			
Name of Microorganisms	1st	2nd	3rd	4th	1st	2nd	3rd	4th	1st	2nd	3rd	4th	1st	2nd	3rd	4th	1st	2nd	3rd	4th	1st	2nd	3rd	4th	1st	2nd	3rd	4th	1st	2nd	3rd	4th	1st	2nd	3rd	4th
Staphylococcus aureus	8.10	8.06	7.63	8.26	4.27	4.10	3.76	4.36	6.89	6.67	6.17	6.08	4.73	3.86	2.64	0.00	6.87	7.73	6.25	6.77	5.30	4.76	4.48	5.88	4.46	4.13	3.48	4.40	5.23	5.32	4.65	5.06	6.23	6.12	5.92	6.22
Influenza B virus	5.51	6.08	5.43	5.63	5.04	6.88	5.81	5.31	5.58	7.48	6.03	5.63	6.71	9.45	8.30	7.07	3.82	4.63	3.39	3.61	2.93	3.32	2.67	3.24	4.06	5.50	4.61	4.61	4.23	5.13	3.80	4.74	4.55	5.46	4.62	4.71
Rubella virus	6.68	6.46	6.56	6.60	4.67	4.61	4.46	4.44	7.82	7.73	7.74	7.86	4.44	4.93	4.40	4.39	7.11	7.17	5.39	4.91	5.94	5.64	5.66	0.86	2.66	2.62	2.21	3.17	3.23	3.38	3.53	3.67	3.56	3.41	3.24	3.35
Influenza A virus H11N6 (A/duck/England/1/1956)	5.32	6.10	5.77	5.61	5.00	7.61	5.83	5.46	6.76	8.43	7.98	6.97	5.87	8.77	8.21	6.41	2.71	3.38	3.55	3.50	4.82	4.89	4.78	4.00	3.82	4.25	4.19	4.19	4.60	5.58	4.51	4.68	3.58	4.70	3.65	3.91
Meyerozyma guilliermondii	3.28	3.86	3.35	3.52	4.66	4.74	4.14	4.79	4.32	4.22	4.62	4.88	0.00	0.00	0.00	0.00	3.10	3.25	3.92	4.13	4.61	4.36	4.49	3.73	3.91	2.82	3.18	2.77	0.00	0.00	0.00	0.00	3.19	3.16	2.87	3.50
Influenza B virus	4.54	4.90	4.68	4.63	4.17	5.97	4.67	4.43	4.52	6.13	4.98	4.61	5.73	8.28	7.09	5.91	2.85	3.68	2.73	3.00	2.06	2.57	1.85	2.43	3.51	4.66	3.92	3.90	3.52	4.35	3.02	4.03	3.78	4.68	3.78	3.85
Legionella cardiaca	1.26	1.11	0.99	1.44	3.89	3.63	4.06	3.86	3.49	3.70	3.56	3.36	1.16	1.31	1.26	1.13	1.30	1.29	0.82	0.39	0.49	0.77	0.78	2.69	0.33	0.36	0.31	0.15	3.42	3.48	3.36	3.73	1.53	1.52	1.33	1.59
Francisella philomiragia	6.04	6.99	6.94	6.00	2.31	2.54	2.83	2.20	6.50	6.78	6.61	6.49	7.35	7.49	7.24	7.43	5.53	5.61	4.74	4.47	4.47	4.41	4.42	0.63	1.65	1.76	1.36	1.91	3.21	3.32	3.25	3.42	5.01	4.89	4.87	4.97
Rubella virus	5.91	5.97	6.03	6.18	4.00	4.06	4.21	4.20	7.41	7.51	7.16	7.33	4.26	4.51	4.50	4.10	6.95	6.76	5.14	4.66	5.53	5.47	5.45	0.67	2.43	2.56	1.94	3.17	3.37	3.20	3.01	3.66	3.50	3.55	2.89	3.63
Influenza A virus H7N9 (A/Anhui/1/2013)	4.76	5.18	5.08	4.79	4.50	6.05	5.16	4.75	5.14	6.49	5.83	5.34	5.46	6.90	6.55	5.53	2.50	2.92	2.14	1.81	3.71	3.72	3.80	2.78	1.91	2.71	2.23	2.43	2.72	3.40	2.65	3.06	3.00	3.57	3.03	3.13

Changes in the level of antibodies against various mirioorganisms with highest SHAP values over time. In the Table, 1, 2, 3 and 4 represent the first, second, third and fourth blood sample collections, respectively. The fluorescence intensity ratios of relative values against the negative controls are indicated as relative log_2 ratios and represented by the red intensity in the figure.
